# Pillars and Pitfalls of the New Pharmacovigilance Legislation: Consequences for the Identification of Adverse Drug Reactions Deriving From Abuse, Misuse, Overdose, Occupational Exposure, and Medication Errors

**DOI:** 10.3389/fphar.2018.00611

**Published:** 2018-06-12

**Authors:** Maurizio Sessa, Gabriella di Mauro, Annamaria Mascolo, Concetta Rafaniello, Liberata Sportiello, Cristina Scavone, Annalisa Capuano

**Affiliations:** ^1^Campania Pharmacovigilance and Pharmacoepidemiology Regional Centre, Section of Pharmacology “L. Donatelli”, Department of Experimental Medicine, University of Campania “L. Vanvitelli”, Naples, Italy; ^2^Department of Drug Design and Pharmacology, University of Copenhagen, Copenhagen, Denmark

**Keywords:** medication errors, spontaneous reporting system, pharmacovigilance, abuse, overdose, occupational exposure, misuse

## Abstract

**Rationale:** The aim of this study is to investigate if following the implementation of the Regulation EU/1235/2010 and the Directive 2010/84/EU there was an increase of individual case safety reports (ICSRs) deriving from a medication error, abuse, misuse, overdose, or occupational exposure. Other objectives are the identification of drugs mostly involved in such cases, to establish if the codification of aforementioned conditions is performed correctly and, whenever codification errors exist, to identify predictors of codification errors. Finally, we estimated the magnitude of these errors on signal detection activities.

**Methods:** ICSRs sent through Campania Region (Italy) spontaneous reporting system from July 2nd 2012 to December 31th 2017 were used as data source. A multivariable logistic regression model was used to identify predictors of codification errors. Four measures of disproportionality were used to investigate the magnitude of codification errors on a known safety signal or rather the association between benzodiazepines derivatives and abuse.

**Results:** In all, 358 (1.4%) out of 25610 ICSRs reported “non-normal use” of drugs, mainly as cases of abuse. Drugs mostly involved in abuse were “Benzodiazepines derivatives” (171/358; 47.8%). For medication errors instead, “Other antiseptics and disinfectants” (9/358; 2.5%). At the first quality control, 125 (34.9%) out of 358 ICSRs did not have a codification of “non-normal use” or codifications were performed wrongly. Codification errors included misclassification of abuse as overdose (10/125; 8.0%) and misclassification of medication error as overdose (7/125; 5.6%) or abuse (7/125; 5.6%). Compared to pharmaceutical companies, patients/citizens (as reporters) had a 24.88 higher odd (Reporting Odds Ratio 24.88, 95%CI 1.82–449.95; *p*-value: 0.02) of performing un-classification or misclassification of aforementioned codifications. Codification errors were associated with the underestimation of measure of disproportionality' estimates in the identification of the safety signal “Benzodiazepine derivatives /abuse”.

**Conclusion:** In conclusion, this study found that in Campania Region (southern Italy) there was an exponential increase of ICSR reporting “non-normal use,” mainly as cases of abuse, with an improvable proportion of cases misclassified/unclassified. Moreover, this study found that ICSRs sent by patients/citizens were associated with an increased odd of un-classification or misclassification that had a relevant impact on signal detection activities.

## Introduction

Despite accomplishments obtained by the European Member States in terms of enhancements of National Pharmacovigilance systems, the European Parliament and the European Council (EC) in December 2010 approved the new Pharmacovigilance legislation or rather the Regulation (EU) No. 1235/2010 and the Directive 2010/84/EU that come into force on July 2nd 2012. These new legal provisions have radically changed the European Pharmacovigilance landscape introducing rules aimed to optimize and speed up the safety signal detection and strengthen the current Pharmacovigilance system by making it more robust and transparent for a better detection, assessment, understanding, and prevention of adverse drug reactions or any other drug-related problem (European Parliament, 2010a,b). With the new Pharmacovigilance legislation, it was introduced a new definition of an adverse event now defined as “any unfavorable and unintended sign (e.g., an abnormal laboratory finding), symptom, or disease temporally associated with the use of a medicinal product” (European Medicine Agency, 2017). With the new definition of an adverse event, it became possible to report those deriving from a medication error, abuse, misuse, overdose, and occupational exposure. According to Good Pharmacovigilance Practices (GVP)—Module VI (Rev 2) an *occupational exposure* “refers to the exposure to a medicinal product, as a result of one's professional or non-professional occupation” and a *misuse* “refers to situations where the medicinal product is intentionally and inappropriately used not in accordance with the authorized product information.” An *abuse*, instead, “corresponds to the persistent or sporadic, intentional excessive use of a medicinal product, which is accompanied by harmful physical or psychological effects” and a *medication error* “refers to any unintentional error in the prescribing, dispensing, or administration of a medicinal product while in the control of the healthcare professional or consumer.” Finally, an *overdose* “refers to the administration of a quantity of a medicinal product given per administration or cumulatively, which is above the maximum recommended dose according to the authorized product information. Clinical judgment should always be applied” (European Medicine Agency, 2017). The European Commission introduced such modifications in the optic of risk minimization aiming to increase their detection to promote their preventability, especially in spontaneous reports or rather spontaneous Individual case safety reports (ICSRs). On a European level, spontaneous ICSRs are collected from consumers (defined as a person who is not a healthcare professional such as a patient, lawyer, friend, relative of a patient or care) and healthcare professional (defined as a medically-qualified person such as a physician, dentist, pharmacist, nurse, coroner, or as otherwise specified by local regulations; European Medicine Agency, 2017). In Italy, consumers and healthcare professionals have three possible ways to report an adverse reaction report. The first option for a reporter is to send the aforementioned report to the local pharmacovigilance manager (local health unit/hospital). The second and third options are to report the adverse reaction report directly to the national competent authority or to the marketing authorization holder, respectively. To the best of our knowledge, to date, in Italy, it is unknown if following the implementation of the Regulation EU/1235/2010 and the Directive 2010/84/EU there was an increase of cases reporting medication errors, abuse, misuse, overdose, or occupational exposure. Even less is known about drugs involved in these cases and if the codification of aforementioned conditions is performed correctly. Additionally, whenever codification errors exist, it is unknown to which extent they could influence signal detection activities. Given these gaps in knowledge, to provide further insight on aforementioned topics we investigated all adverse events deriving from medication errors, abuse, misuse, overdose, and occupational exposure among those reported through Campania Region (an Italian Region of southern Italy with 6 million citizens) spontaneous reporting system from July 2nd 2012 to December 31th 2017.

## Methods

### Study design

Passive pharmacovigilance project that includes a case series of ICSRs reporting abuse, misuse, overdose, occupational exposure and medication errors (World Health Organization, 2012).

### Data source

We retrieved from the Italian National database for Pharmacovigilance (*Rete Nazionale di Farmacovigilanza*, RNF) all ICSRs reported through Campania Region spontaneous reporting system from July 2nd 2012 to December 31th 2017. The Italian Medicine Agency (AIFA) manages RNF that stores all ICSRs of suspected adverse drug reactions that have been reported on the National territory. Before being recorded in RNF, Local Pharmacovigilance Managers (LPM) of local health units, hospitals, and Scientific Institutes for Hospitalization and Care (IRCCS) evaluates the quality of information reported in ICSRs and retrieved missing information needed for a proper causality assessment.

### Case-by-case assessment

For each ICSR sent through Campania Region spontaneous reporting system and recorded in RNF, Campania Pharmacovigilance and Pharmacoepidemiology Regional Centre (CPPRC) validates data included in ICSRs and performs causality assessment for all drug-event couples using the Naranjo algorithm. If discrepancies were identified, CPPRC proceeds to reconciliation with both LPM and the reporter (through LPM).

### Validation of abuse, overdose, misuse, medication error, and occupational exposure codifications in ICSRS

From July 2nd 2012, for the purpose of this study, CPPRC recorded in an *ad-hoc* database all ICSRs for which a codification of abuse, overdose, misuse, medication errors, and occupational exposure was needed. A codification was considered as needed if personnel of CPPRC, by re-evaluating the ICSR, found that the case was classifiable as abuse, overdose, misuse, medication errors, or accidental exposure as in accordance with the Good Pharmacovigilance Practice (European Medicine Agency, 2017). We considered a codification of abuse, overdose, misuse, medication error, and occupational exposure as assigned erroneously if it was not in accordance with a forementioned Pharmacovigilance guidelines (European Medicine Agency, 2017).

### Statistical analyses

To provide an overview of the number of cases of abuse, overdose, misuse, medication errors and occupational exposure reported in Campania Region along with drugs reported as suspected in these cases, we plotted the number of ICSRs stratified by year. Drugs were grouped in chemical subgroup according to the fourth level of the Anatomical Therapeutic Chemical (ATC) classification system. Codification errors along with representative examples were tabled. The number of codification errors for abuse, overdose misuse, medication error, and occupational exposure was stratified by year and it was plotted. The percentage distributions of ADRs [grouped by System Organ Class (SOC)] was plotted singularly for abuse, misuse, medication error, and occupational exposure. For descriptive purposes, clinical and demographic characteristics of cases, seriousness, and outcome of ADRs and the type of reporter stratified by abuse, overdose, misuse, medication error, and occupational exposure were reported. Based on the ICSR form available on the National territory, for the outcome, six categories were used: resolution with sequelae, unchanged clinical condition, recovered, improvement, death, and not available. Seriousness was codified according to the International Council on Harmonization E2D guidelines (International Conference on Harmonisation E2D, 2018). A multivariable logistic regression model was used to identify predictors of codification errors among the year of reporting, age, gender, the reporter qualification, suspected drugs (grouped as shown in Supplementary Table 1), and ADRs (classified by SOC, as shown in Supplementary Table 2). In particular, the reporting odds ratio (ROR) with 95% confidence intervals (95%CI) was computed (Rothman et al., 2004). For this analysis, we considered a case as having a codification error if a codification was not included (but it was needed) and if the codification was assigned erroneously. To investigate the magnitude of abuse' codification errors on signal detection activities (abuse was chosen because it was the most representative “non-normal use” condition in ICSRs), we investigated a known safety signal, or rather the association between benzodiazepines derivatives and abuse (O'brien, 2005). In particular, for the aforementioned safety signal, we tested four measures of disproportionalities and in particular the Proportional Reporting Ratio (PRR), the Reporting Odds Ratio (ROR), the Relative Reporting Ratio (RRR), and the Information Component (IC) with and without correcting codification errors (Wilson et al., 2004; Van Holle and Bauchau, 2014; Böhm et al., 2016). The PRR, ROR, RRR, and IC were computed as shown in Table 1.

**Table 1:** Operative definitions of measures of disproportionality.

$PRR=\frac{A}{A+B}/\frac{C}{C+D}$ $ROR=\frac{A}{B}/\frac{C}{D}$ *RRR* = *A* * (*A* + *B* + *C* + *D*)/(*A* + *B*) * (*A* + *C*) $IC=\;log_{2}\frac{p\left(x,y\right)}{p\left(x\right)p\left(y\right)}$	**Where:**		
		**Abuse**	**All other events**
	**Benzodiazepine derivatives**	A	B
	**All other drugs**	C	D
	**Where:**		
	**p(x)** = probability that benzodiazepine derives listed on ICSR;		
	**p(y**) = probability that abuse listed on ICSR;		
	**p(y,x)** = probability that benzodiazepine derivatives-abuse combination listed on ICSR.		

*PRR, Proportional Reporting Ratio; ROR, Reporting Odds Ratio; RRR, Relative Reporting Ratio; IC, Information Component*.

## Results

### Overview of ICSRs deriving from a medication error, abuse, misuse, overdose, or occupational exposure among those reported through campania region spontaneous reporting system

**Figure 1:**
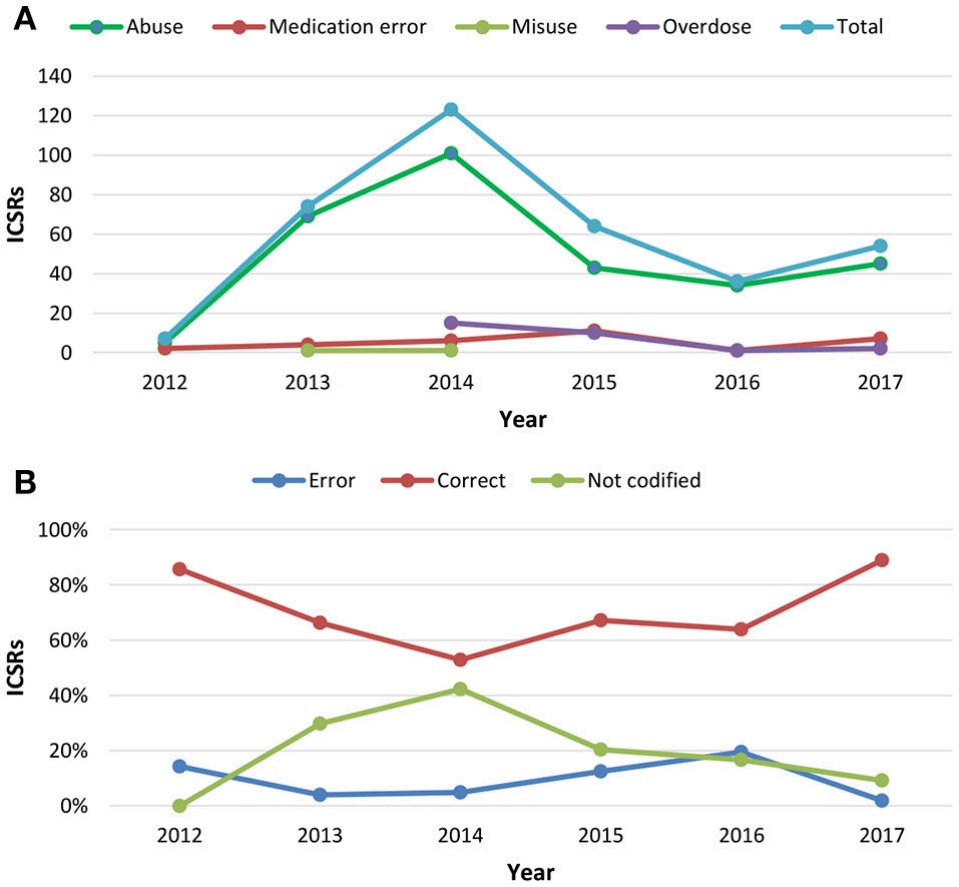
**(A)** Trend of Individual Case Safety Reports (ICSRs) deriving from abuse, medication error, misuse and overdose reported in Campania Region spontaneous reporting system from July 2nd 2012 to December 31th 2017; **(B)** percentage distribution trend of un-classification or misclassification of cases identified using the same data source in the same study period.

**Figure 2:**
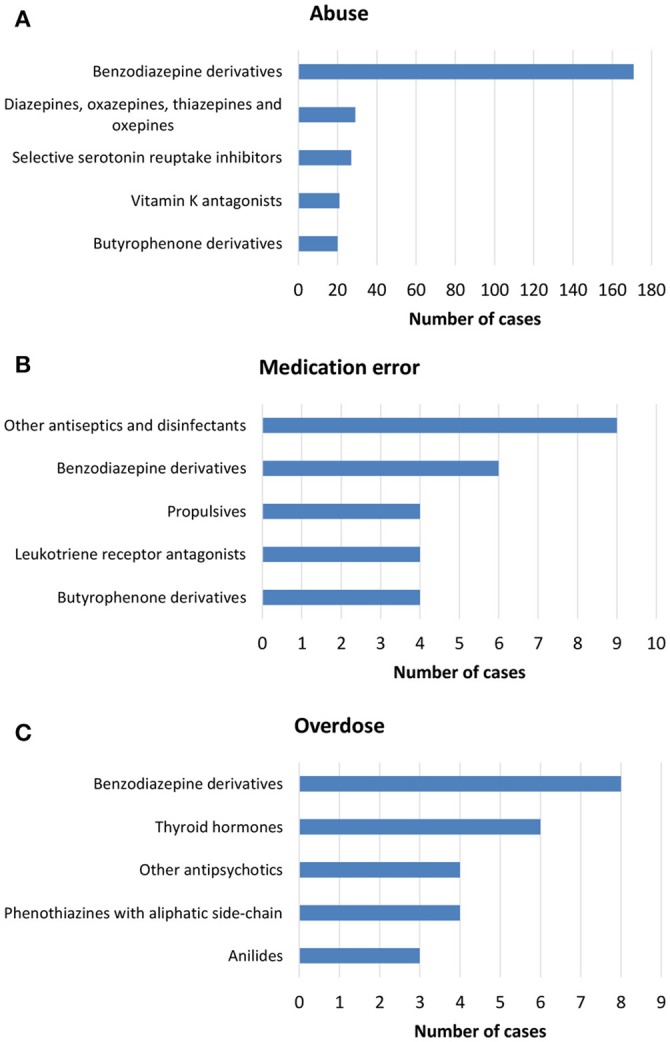
Top-five drug classes reported in ICSRs deriving from **(A)** abuse, **(B)** medication error, and **(C)** overdose that were sent through Campania Region spontaneous system from July 2nd 2012 to December 31th 2017.

**Figure 3:**
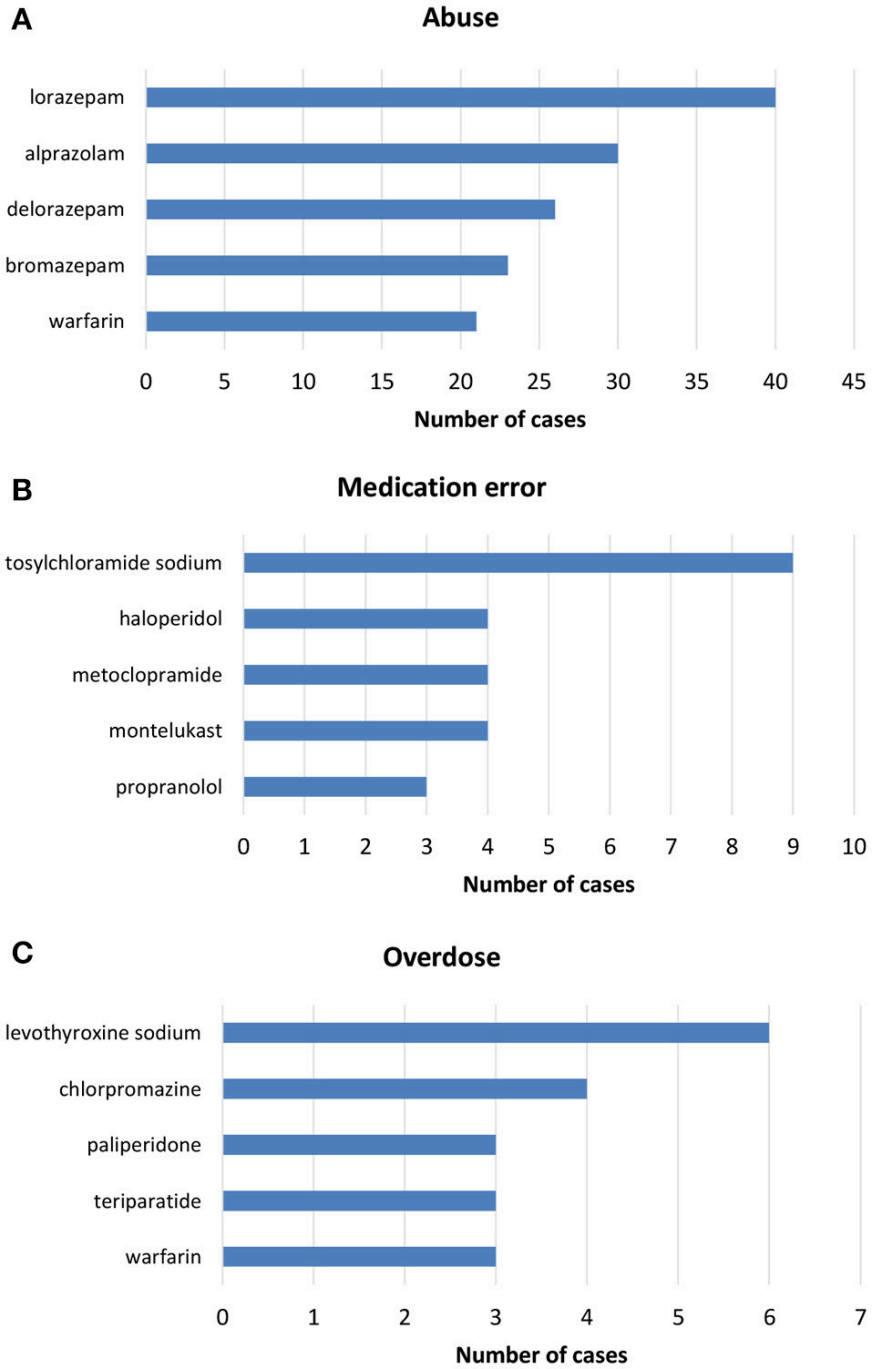
Top-five drugs reported in ICSRs deriving from **(A)** abuse, **(B)** medication error, and **(C)** overdose that were sent through Campania Region spontaneous system from July 2nd 2012 to December 31th 2017.

**Figure 4:**
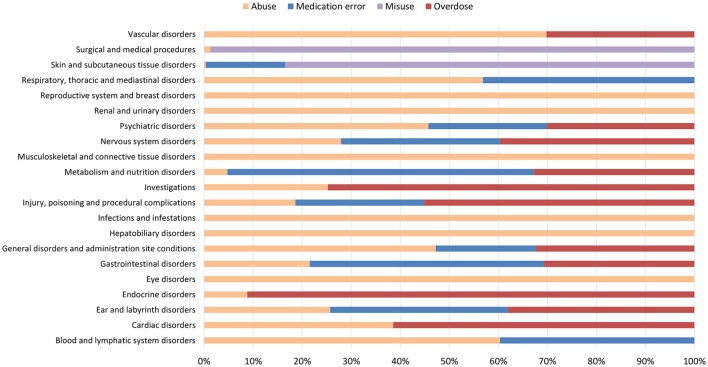
Percentage distribution of adverse drug reactions (classified by System Organ Class) reported in ICSRs deriving from abuse, misuse, medication error and occupational exposure that were sent through Campania Region spontaneous system from July 2nd 2012 to December 31th 2017.

From July 2nd 2012 to December 31th 2017, 358 (1.4%) out of 25610 ICSRs reported abuse, overdose, misuse, medication error, or occupational exposure among those sent through Campania Region spontaneous reporting system (Table 2, Figure 1, Supplementary Figures 1–5). Drugs mostly involved in abuse were “Benzodiazepines derivatives” (171/358; 47.8%) and in particular alprazolam (40/171; 23.4%), lorazepam (30/171; 17.5%), and delorazepam (26/171; 15.2%). For medication errors instead, “Other antiseptics and disinfectants” (9/358; 2.5%) with all cases involving tosylchloramide sodium (9/9; 100.0%) (Figures 2, 3). Percentage distribution of adverse drug reactions (classified by SOC) distributed by abuse, overdose, misuse, medication error, and occupational exposure was provided in Figure 4. At the first quality control by CPPRC, 125 (34.9%) out of 358 ICSRs did not have a codification of abuse, overdose, misuse, medication error and occupational exposure or codifications were performed erroneously (Figure 1). Errors that were more frequently identified in ICSRs included misclassification of cases of abuse as overdose (10/125; 8.0%) and misclassification of medication errors as overdose (7/125; 5.6%) or abuse (7/125; 5.6%) (Table 3).

**Table 2:** Demographic characteristics, seriousness, and reporter qualification of ICSRs deriving from abuse, misuse, medication error and occupational exposure recognized in Campania Region spontaneous reporting system from July 2nd 2012 to December 31th 2017.

**Variable**	**Level**	**Abuse (*n* = 297)**	**Medication error (*n* = 31)**	**Misuse (*n* = 2)**	**Overdose (*n* = 28)**	**Total (*n* = 358)**
Gender	Female	198 (66.7)	17 (54.8)	2 (100.0)	18 (64.3)	235 (65.6)
	Male	99 (33.3)	14 (45.2)	0 (0.0)	10 (35.7)	123 (34.4)
Age	mean (SD)	43.9 (18.9)	35.0 (32.1)	67.0 (15.6)	37.8 (24.1)	42.7 (20.9)
Seriousness	Not defined	7 (2.4)	1 (3.2)	0 (0.0)	1 (3.6)	9 (2.5)
	Not serious	96 (32.3)	15 (48.4)	1 (50.0)	18 (64.3)	130 (36.3)
	Serious - death	1 (0.3)	0 (0.0)	0 (0.0)	0 (0.0)	1 (0.3)
	Serious - life threatening	23 (7.7)	1 (3.2)	0 (0.0)	0 (0.0)	24 (6.7)
	Serious - other clinically relevant condition	71 (23.9)	1 (3.2)	0 (0.0)	4 (14.3)	76 (21.2)
	Serious - hospitalization	99 (33.3)	13 (41.9)	1 (50.0)	5 (17.9)	118 (33.0)
Reporter	Anti-poison center	8 (2.7)	4 (12.9)	0 (0.0)	1 (3.6)	13 (3.6)
	Hospital physician	173 (58.2)	19 (61.3)	0 (0.0)	12 (42.9)	204 (57.0)
	Other healthcare professionals	31 (10.4)	2 (6.5)	1 (50.0)	4 (14.3)	38 (10.6)
	Patients/citizens	2 (0.7)	1 (3.2)	0 (0.0)	4 (14.3)	7 (2.0)
	Pharmaceutical companies	8 (2.7)	1 (3.2)	0 (0.0)	0 (0.0)	9 (2.5)
	Pharmacist	75 (25.3)	4 (12.9)	1 (50.0)	7 (25.0)	87 (24.3)

*SD, standard deviation*.

**Table 3:** Representative examples of codification errors for cases deriving from abuse, misuse, medication error, and occupational exposure.

**Codification at the first ICSR evaluation**	**Final codification following a case-by-case re-valuation**	**Representative examples of codification errors**	**Number of ICSRs**
Overdose	Abuse	i.e., attempted suicides	10
Overdose	Medication error	i.e., unintentional administration/use	7
Abuse	Medication error	i.e., unintentional administration/use	7
Abuse	Misuse	i.e., intentional use a topical solution per os	2

*Individual Case Safety Report (ICSR)*.

### Predictors of un-classification/misclassification of abuse, overdose, misuse, medication error and occupational exposure

**Figure 5:**
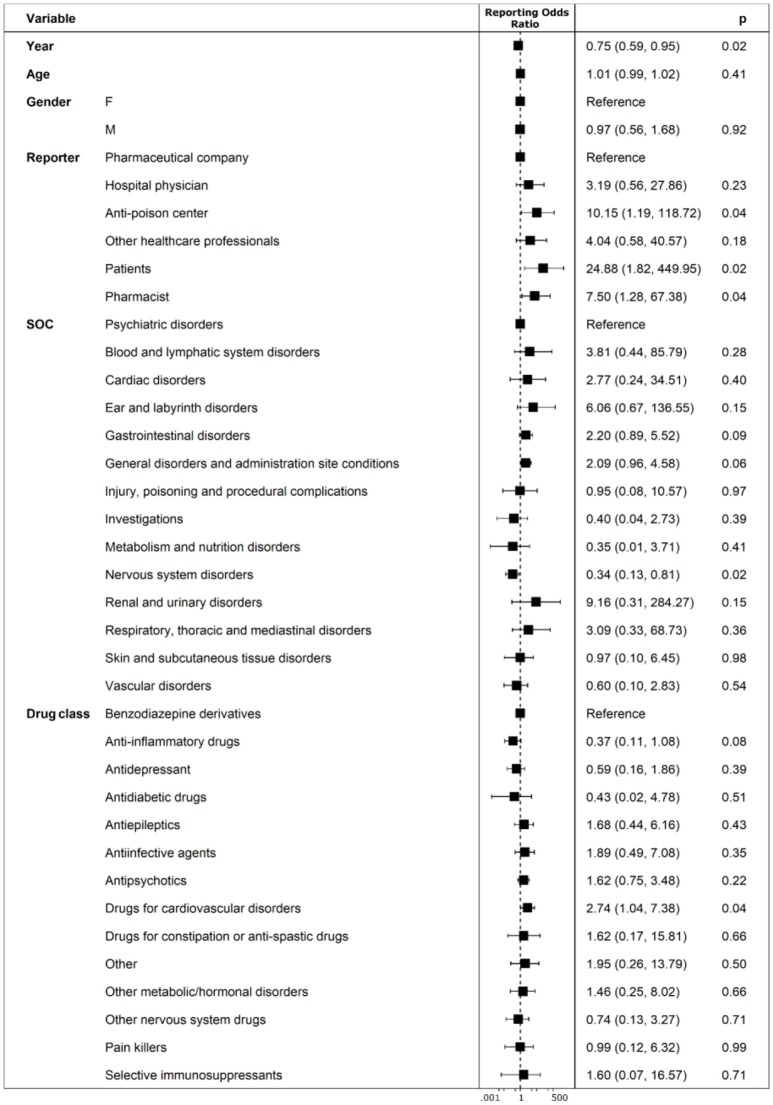
Predictors of un-classification or misclassification of ICSRs deriving from abuse, misuse, medication error and occupational exposure that were sent through Campania Region spontaneous system from July 2nd 2012 to December 31th 2017.

Years following the introduction of the Directive 2010/84/EU and the Regulation (EU) No 1235/2010 were found protective for un-classification or misclassification of abuse, overdose, misuse, medication error, and occupational exposure. Such errors reduced from July 2nd 2012 to December 31th 2017 with a statistically significant lower probability of having un-classification or misclassification of 25% for each year subsequent to 2012 (ROR 0.75, 95%CI 0.59–0.95; *p*-value: 0.02). Compared to pharmaceutical companies for which no codification errors were identified, patients/citizens (as reporters) had a 24.88 higher odd (ROR 24.88, 95%CI 1.82–449.95; *p*-value: 0.02) of performing un-classification or misclassification of abuse, overdose, misuse, medication error and occupational exposure. Similarly, anti-poison center and pharmacists (as reporters) had a 10.15 (ROR 10.15, 95%CI 1.19-118.72; *p*-value: 0.04) and 7.50 higher odd (ROR 7.50, 95%CI 1.28-67.38; *p*-value: 0.04) of performing aforementioned errors, respectively. For ICSRs having adverse drug reactions classified as “Nervous system disorders,” such as coma, asthenia, sleepiness and psychomotor agitation, it was found a 66% lower odd (ROR 0.34, 95%CI 0.13-0.81; *p*-value: 0.02) of having un-classification or misclassification. ICSRs that reported “drugs for cardiovascular disorders” as suspected (mainly vitamin K antagonist and β-blockers) were found to have a 2.74 higher odd (ROR 2.74, 95%CI 1.04-7.38; *p*-value: 0.04) of un-classification or misclassification if compared to “Benzodiazepine derivatives” (Figure 5).

### Impact of codification errors on signal detection activities

**Figure 6:**
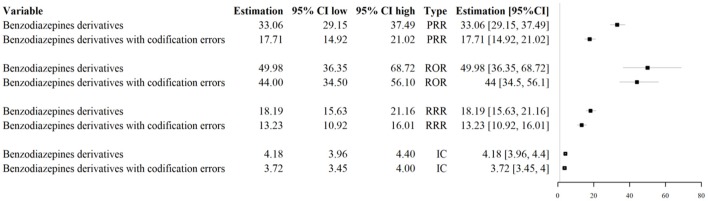
Impact of codification errors on signal detection activities for the signal “Benzodiazepine derivatives/abuse.” *PRR, Proportional Reporting Ratio; ROR, Reporting Odds Ratio; RRR, Relative Reporting Ratio; IC, Information Component*.

Codification errors were associated with the underestimation of PRR, ROR, RRR, and IC' estimates in the identification of the safety signal “Benzodiazepine derivatives/abuse” (Figure 6).

## Discussion

This study is a set of initiatives proposed by CPPRC to promote drug safety (Parretta et al., 2014; Mascolo et al., 2016; Rafaniello et al., 2016; Sessa et al., 2016a,c, 2017a). Aforementioned initiatives include strengthening of the Regional pharmacovigilance system by introducing more clearly defined roles and responsibilities of stakeholders. Moreover, the early identification of preventable adverse drug reactions occurring on the Regional territory and the promotion of an appropriate use of medicinal products. By revaluating all ICSRs sent through Campania Region spontaneous reporting system from July 2nd 2012, we found an exponential increase of ICSR reporting abuse, overdose, misuse, medication error and occupational exposure in the first 2 years following the implementation of the Regulation EU/1235/2010 and the Directive 2010/84/EU. The majority of cases derived from a condition of abuse with “Benzodiazepines derivatives” and in particular alprazolam, lorazepam, and delorazepam. Our results are in line with those obtained by Leporini and colleagues in Calabria Region (southern Italy), despite aforementioned authors obtained such results from an active pharmacovigilance project and, therefore, using a different methodology (Leporini et al., 2017). In fact, as opposed to Leporini and colleagues, we performed a passive Pharmacovigilance project that focused exclusively on the identification of ICSRs deriving from the non-normal use of medical products. Our study, provide a detailed description of demographic and clinical characteristics of aforementioned cases and a detailed revision of the most reported suspected drugs and adverse drug reactions for each non-normal condition of use. It should be highlighted that as opposed to Leporini et al., we performed a case-by-case validation of all ICSRs in order to re-assess “non-normal” conditions in ICSRs. In fact, we did not rely on codifications that were performed prior to the data recording in RNF. Despite aforementioned differences, in accordance with our results, Leporini and colleagues observed that the majority of cases derived from abuse, mainly with benzodiazepines. In this regard, it is not surprising to find benzodiazepines derivatives in cases of abuse. In fact, for our Region, we previously described such phenomenon (Sessa et al., 2016b) and, in the scientific community, it is widely accepted that benzodiazepines derivatives usage could be associated with abuse, self-harm or attempt suicide (Longo and Johnson, 2000; Neale and Smith, 2007). For medication errors instead, we found that the “Other antiseptics and disinfectants” and in particular topical solutions of tosylchloramide sodium, was the most reported in such cases. For our Country (Italy), this phenomenon was described in the scientific literature. In fact, in the Italian pharmaceutical market there are liquid formulations of tosylchloramide sodium for disinfection of skin' wounds or external genitalia. Aforementioned formulations are granules that need to be dissolved in water. Being such formulations similar to granulates of other active ingredients that are supposed to be used orally, patients tends to get confused ingesting the topic solution of tosylchloramide sodium orally (Lariccia et al., 2013). By looking at the percentage distribution of adverse drug reactions, as expected, the most representative were “type A” adverse drug reactions (or rather dose-dependent adverse drug reactions according to Rawlins and Thompson classification Rawlins, 1977). This result should be considered in virtue of abuse/overdose as the most reported “non-normal use” condition in Campania Region spontaneous reporting system. Interestingly, 1 out of 3 ICSRs deriving from abuse, misuse, medication error, and occupational exposure did not have a codification or codifications were performed erroneously. Errors were frequent during the first 2 years following the introduction of the new Pharmacovigilance legislation however, with a statistically significant reduction over time, especially in the last 2 years. In this regards, we believe that a great impact for this result was played by education activities performed by CPPRC and AIFA on both the Regional and National territory (Sessa et al., 2016b,d, 2017b). Among predictors for codification errors, ICSRs sent by patients/citizens were found associated with an increased odd of un-classification or misclassification of abuse, overdose, misuse, medication error, and occupational exposure. Whenever confirmed with additional studies, our result opens a “Pandora box” in terms of potential differences in the quality of ICSRs sent by patients/citizens. In fact, we proved that in contrast with what was previously found for ICSRs reporting “normal” use of drugs (Rolfes et al., 2017), the quality of ICSRs sent by patients could be lower than healthcare professionals when there is the need to codify “non-normal” conditions (Rolfes et al., 2017). Analogously, a crucial aspect of our results is that anti-poison center and pharmacists' error trend did not overlap with the general trend. In fact, anti-poison center and pharmacists showed a progressive reduction of the correct codification. In this regard, we believe that corrective measures are necessary. In fact, we will organize *ad-hoc* courses to train anti-poison center and pharmacists on the proper codification of adverse drug reactions deriving from the non-normal use of medicinal products. Additionally, in our study, we found specific clinical scenarios for which it was more likely to perform a codification error. In particular, ICSRs that reported “drugs for cardiovascular disorders” as suspected (mainly vitamin K antagonist and β-blockers) were found to have a 2.74 higher odd of un-classification or misclassification if compared to cases reporting “Benzodiazepine derivatives.” In these cases, codification errors mainly involved misclassification of abuse or overdose. We believe that a possible explanation for aforementioned errors (but more generally for all codification errors involving abuse/overdose) could be the actual definitions of abuse/overdose. If we imagine a clinical scenario where a patient treated with vitamin K antagonist use an excessive dose of this drug for attempting suicide and we look at the current definition of overdose and abuse, both definitions partially cover this scenario making hard the choice of the correct codification for non-trained reporter. In particular, for the definition of overdose “…*administration of a quantity of a medicinal product given per administration or cumulatively, which is above the maximum recommended dose*” while for the definition of abuse “…*intentional excessive use of a medicinal product, which is accompanied by harmful physical or psychological effects*” (European Medicine Agency, 2017). Additionally, we believe that a cause for aforementioned errors could be the inconsistency between the new “regulatory” definition of “abuse/overdose” and the currently available definitions (i.e., pharmacological, psychiatric, etc.) for which there is a lack of consensus (Rinaldi et al., 1988; World Health Organization, 1994; Substance Abuse and Mental Health Services Administration, 2001; Cicero et al., 2007; Katz et al., 2007; Bronstein et al., 2008). Finally, another interesting result of our study is the quantification of the underestimation of PRR, ROR, RRR, and IC' estimates due to codification errors while identifying the known safety signal “Benzodiazepine derivatives/abuse.” In particular, independently of the measure of disproportionality used, codification errors were associated with an underestimation of disproportionality measures' estimates. This effect was statistically significant for PRR (as confidence intervals with and without considering codification errors did not overlap), while it was statistically insignificant for ROR, RRR, and IC. In this regard, it could not be excluded that whenever measures to minimize codification errors will not be implemented, these errors might play a key role in masking new safety signal for which magnitude will not be massive as for the safety signal “Benzodiazepine derivatives/abuse.” In this regard, we believe that to mitigate aforementioned errors might be beneficial to include “attempted suicide” among “non-normal use” scenarios.

## Conclusion

In conclusion, this study found that in Campania Region (southern Italy) there was an exponential increase of ICSR reporting abuse, overdose, misuse, medication error and occupational exposure following the implementation of the new Pharmacovigilance legislation with an improvable proportion of cases misclassified/unclassified. Moreover, this study found that ICSRs sent by patients/citizens were associated with an increased odd of un-classification or misclassification and that un-classification or misclassification had a relevant impact on signal detection activities. Educational activities will be organized to guarantee the correct codification of abuse, misuse, overdose, medication error, and occupational exposure on the Regional territory.

## Datasets are available on request

The raw data supporting the conclusions of this manuscript will be made available by the authors, without undue reservation, to any qualified researcher.

## Author contributions

MS Developed the concept and designed the study. MS, GdM, and AM Analysis, or interpretation of data. MS, GdM, AM, CR, LS, CS, AC Drafting the paper and revising it for important intellectual content. MS Wrote the paper. MS, GdM, AM, CR, LS, CS, and AC Final approval of the version to be published.

### Conflict of interest statement

The authors declare that the research was conducted in the absence of any commercial or financial relationships that could be construed as a potential conflict of interest.

## Supplementary material

Supplementary Table 1 Grouping of suspected drugs identified in ICSRs sent through Campania Region (southern Italy) spontaneous reporting system from July 2nd 2012 to December 31th 2017.

Supplementary Table 2 Classification by System Organ Class of adverse drug reactions identified in ICSRs sent through Campania Region (southern Italy) spontaneous reporting system from July 2nd 2012 to December 31th 2017.

Supplementary Figure 1 Trend of Individual Case Safety Reports (ICSRs) deriving from abuse reported in Campania Region spontaneous reporting system from July 2nd 2012 to December 31th 2017.

Supplementary Figure 2 Trend of Individual Case Safety Reports (ICSRs) deriving from medication error reported in Campania Region spontaneous reporting system from July 2nd 2012 to December 31th 2017.

Supplementary Figure 3 Trend of Individual Case Safety Reports (ICSRs) deriving from overdose reported in Campania Region spontaneous reporting system from July 2nd 2012 to December 31th 2017.

Supplementary Figure 4 Trend of Individual Case Safety Reports (ICSRs) deriving from misuse reported in Campania Region spontaneous reporting system from July 2nd 2012 to December 31th 2017.

Supplementary Figure 5 Percentage distribution trend of un-classification or misclassification of cases reported by anti-poison center and pharmacists that were identified in Campania Region spontaneous reporting system from July 2nd 2012 to December 31th 2017.
