# Supplementary material for: Pillars and Pitfalls of the New Pharmacovigilance Legislation: Consequences for the Identification of Adverse Drug Reactions Deriving From Abuse, Misuse, Overdose, Occupational Exposure, and Medication Errors
Source: Front Pharmacol. 2018 Jun 12;9:611. doi: 10.3389/fphar.2018.00611 (PMC6006791; doi:10.3389/fphar.2018.00611)
Supplement: Supplementary Table 1 — Grouping of suspected drugs identified in ICSRs sent through Campania Region (southern Italy) spontaneous reporting system from July 2nd 2012 to December 31th 2017. [file Table_1.docx]

Supplementary Material

Pillars and pitfalls of the new Pharmacovigilance legislation: consequences for the identification of adverse drug reactions deriving from abuse, misuse, overdose, occupational exposure and medication errors.

Maurizio Sessa^1,2*⸸^ & Gabriella di Mauro^1⸸^, Annamaria Mascolo^1^, Concetta Rafaniello^1^, Liberata Sportiello^1^, Cristina Scavone^1**^& Annalisa Capuano^1**^

^1^Campania Pharmacovigilance and Pharmacoepidemiology Regional Centre, Section of Pharmacology “L. Donatelli”, Department of Experimental Medicine, University of Campania “L. Vanvitelli”, Naples, Italy

^2^Department of Drug Design and Pharmacology, University of Copenhagen, Copenhagen, Denmark

**^⸸^** Equally credited first authors

** Equally credited last authors

*** Correspondence:**Maurizio Sessa
[maurizio.sessa@unicampania.it](mailto:maurizio.sessa@unicampania.it)
maurizio.sessa@sund.ku.dk

**Supplementary table 1.** Grouping of suspected drugs identified in ICSRs sent through Campania Region (southern Italy) spontaneous reporting system from July 2st 2012 to December 31th 2017.

| **Antidepressant** |
| --- |
| Non-selective monoamine reuptake inhibitors |
| Other antidepressants |
| Selective serotonin reuptake inhibitors |
| **Antidiabetic drugs** |
| Biguanides |
| Combinations of oral blood glucose lowering drugs |
| Dipeptidyl peptidase 4 (DPP-4) inhibitors |
| Insulins and analogues for injection, fast-acting |
| **Antiepileptics** |
| Other antiepileptics |
| **Antiinfectives agents** |
| Antiinfectives and antiseptics for local oral treatment |
| Benzimidazole derivatives |
| Combinations of penicillins, incl. beta-lactamase inhibitors |
| Mercurial products |
| Other antiseptics and disinfectants |
| Penicillins with extended spectrum |
| Third-generation cephalosporin |
| Triazole derivatives |
| **Anti-inflammatory drugs** |
| Acetic acid derivatives and related substances |
| Anilides |
| Corticosteroids, weak (group I) |
| Coxibs |
| Fenamates |
| Other anti-inflammatory and antirheumatic agents, non-steroids |
| Propionic acid derivatives |
| Salicylic acid and derivatives |
| **Antipsychotics** |
| Benzamides |
| Butyrophenone derivatives |
| Diazepines, oxazepines, thiazepines and oxepines |
| Lithium |
| Other antipsychotics |
| Phenothiazines with aliphatic side-chain |
| Phenothiazines with piperazine structure |
| Synthetic anticholinergic agents in combination with psycholeptics |
| Tertiary amines |
| **Benzodiazepine derivatives** |
| Benzodiazepine derivatives |
| Benzodiazepine related drugs |
| Other hypnotics and sedatives |
| **Drugs for cardiovascular disorders** |
| ACE inhibitors and diuretics |
| ACE inhibitors, plain |
| Angiotensin II antagonists, plain |
| Antiarrhythmics, class III |
| Beta blocking agents, non-selective |
| Beta blocking agents, selective |
| Digitalis glycosides |
| Dihydropyridine derivatives |
| Direct thrombin inhibitors |
| Fatty acid derivatives |
| HMG CoA reductase inhibitors |
| Organic nitrates |
| Selective beta-2-adrenoreceptor agonists |
| Thiazides, plain |
| Vitamin K antagonists |
| **Drugs for constipation or anti-spastic drugs** |
| Antispastics |
| Other intestinal adsorbents |
| Propulsives |
| **Other** |
| Aminoquinolines |
| Leukotriene receptor antagonists |
| Other antihistamines for systemic use |
| Other antivirals |
| Other drugs affecting bone structure and mineralization |
| Progestogens and estrogens, fixed combinations |
| **Other metabolic/hormonal disorders** |
| Parathyroid hormones and analogues |
| Preparations inhibiting uric acid production |
| Thyroid hormones |
| **Other nervous system drugs** |
| Anticholinesterases |
| Antivertigo preparations |
| Carboxamide derivatives |
| Drugs used in alcohol dependence |
| Drugs used in erectile dysfunction |
| Drugs used in opioid dependence |
| Other anti-dementia drugs |
| Other centrally acting agents |
| Other nervous system drugs |
| **Pain killers** |
| Other opioids |
| **Selective immunosuppressant** |
| Selective immunosuppressant |
| Tumor necrosis factor alpha (TNF-α) inhibitors |
