# Supplementary material for: Pillars and Pitfalls of the New Pharmacovigilance Legislation: Consequences for the Identification of Adverse Drug Reactions Deriving From Abuse, Misuse, Overdose, Occupational Exposure, and Medication Errors
Source: Front Pharmacol. 2018 Jun 12;9:611. doi: 10.3389/fphar.2018.00611 (PMC6006791; doi:10.3389/fphar.2018.00611)
Supplement: Supplementary Table 2 — Classification by System Organ Class of adverse drug reactions identified in ICSRs sent through Campania Region (southern Italy) spontaneous reporting system from July 2nd 2012 to December 31th 2017. [file Table_2.docx]

Supplementary Material

Pillars and pitfalls of the new Pharmacovigilance legislation: consequences for the identification of adverse drug reactions deriving from abuse, misuse, overdose, occupational exposure and medication errors.

Maurizio Sessa^1,2*⸸^ & Gabriella di Mauro^1⸸^, Annamaria Mascolo^1^, Concetta Rafaniello^1^, Liberata Sportiello^1^, Cristina Scavone^1**^& Annalisa Capuano^1**^

^1^Campania Pharmacovigilance and Pharmacoepidemiology Regional Centre, Section of Pharmacology “L. Donatelli”, Department of Experimental Medicine, University of Campania “L. Vanvitelli”, Naples, Italy

^2^Department of Drug Design and Pharmacology, University of Copenhagen, Copenhagen, Denmark

**^⸸^** Equally credited first authors

** Equally credited last authors

*** Correspondence:**Maurizio Sessa
[maurizio.sessa@unicampania.it](mailto:maurizio.sessa@unicampania.it)
maurizio.sessa@sund.ku.dk

**Supplementary table 2.** Classification by System Organ Class of adverse drug reactions identified in ICSRs sent through Campania Region (southern Italy) spontaneous reporting system from July 2st 2012 to December 31th 2017.

| **Blood and lymphatic system disorders** |
| --- |
| anaemia |
| coagulation disorder |
| hemolysis |
| myelosuppression |
| **Cardiac disorders** |
| arrhythmia |
| atrial fibrillation |
| bradycardia |
| long QT |
| palpitation |
| tachycardia |
| **Ear and labyrinth disorders** |
| vertigo |
| **Endocrine disorders** |
| Cushing syndrome |
| hyperthyroidism |
| **Eye disorders** |
| miosis |
| **Gastrointestinal disorders** |
| abdominal cramps |
| abdominal pain |
| abdominal swelling |
| diarrhea |
| dry mouth |
| duodenal ulcer |
| dyspepsia |
| erosive gastritis |
| gastritis |
| gastroesophageal reflux |
| hemorrhage (digestive tract) |
| hematemesis |
| lip edema |
| melena |
| nausea |
| respiratory failure |
| rectorrhagia |
| ulcerative esophagitis |
| upper abdominal pain |
| vomit |
| **General disorders and administration site conditions** |
| asthenia |
| chest pain |
| chest pressure sensation |
| fatigue |
| gait disturbance |
| hyperpyrexia |
| hyperthermia |
| malaise |
| mucositis |
| slow |
| therapeutic ineffectiveness |
| **Hepatobiliary disorders** |
| hepatitis |
| hypertransaminasemia |
| **Infections and infestations** |
| candidiasis |
| subcutaneous abscess |
| **Injury, poisoning and procedural complications** |
| fall |
| overdose |
| poisoning |
| **Investigations** |
| abnormal coagulation profile |
| abnormal glutamic-pyruvic transaminase |
| abnormal heart tones |
| CPK increased |
| extended QT interval |
| hyperthermia |
| increased bilirubin |
| increased blood calcium |
| increased blood creatine phosphokinase MB |
| INR increased |
| long QT |
| drowsiness |
| transaminases increased |
| weight loss |
| **Metabolism and nutrition disorders** |
| hyperglycemia |
| hypoglycemia |
| hypokalemia |
| hypokalemia |
| hyponatremic syndrome |
| lack of appetite |
| **Musculoskeletal and connective tissue disorders** |
| muscle weakness |
| osteonecrosis |
| rhabdomyolysis |
| **Nervous system disorders** |
| aggravated malignant neuroleptic syndrome |
| akathisia |
| amnesia |
| asthenia |
| attention deficit |
| bradykinesia |
| coma |
| convulsions |
| dizziness |
| dysarthria |
| dyskinesia |
| dystonia |
| epilepsy |
| extrapyramidal symptoms |
| headache |
| hypoglycemic coma |
| hyperkinesia |
| language disorder |
| lethargy |
| loss of consciousness |
| obnubilation |
| orthostatic collapse |
| overtone |
| paresthesia |
| parkinsonism |
| pre-syncope |
| psychomotor agitation |
| reduced attention |
| sedation |
| sensory disturbance |
| sleepiness |
| slow language |
| syncope |
| torpor |
| tremor |
| word disorder |
| **Psychiatric disorders** |
| agitation |
| anxiety |
| bipolar disorder |
| confusion |
| delirium |
| depression |
| disorientation |
| drowsiness |
| drug abuse |
| hallucinations |
| hypomania |
| intentional self-harm |
| physical agitation |
| psychomotor retardation |
| restlessness |
| stress |
| suicide attempted |
| **Renal and urinary disorders** |
| kidney failure |
| **Reproductive system and breast disorders** |
| genital discomfort |
| **Respiratory, thoracic and mediastinal disorders** |
| cough |
| dyspnea |
| hemothorax |
| respiratory failure |
| stress dyspnea |
| **Skin and subcutaneous tissue disorders** |
| erythema |
| exanthema |
| hyperhidrosis |
| thinning of the hair |
| **Surgical and medical procedures** |
| administration - related ADR |
| barbiturate related intoxication (NAS) |
| trauma |
| **Vascular disorders** |
| hemorrhage |
| hemorrhagic shock |
| hypotension |
| pallor |
