# Supplementary figures and images for: Pillars and Pitfalls of the New Pharmacovigilance Legislation: Consequences for the Identification of Adverse Drug Reactions Deriving From Abuse, Misuse, Overdose, Occupational Exposure, and Medication Errors
Source: Front Pharmacol. 2018 Jun 12;9:611. doi: 10.3389/fphar.2018.00611 (PMC6006791; doi:10.3389/fphar.2018.00611)

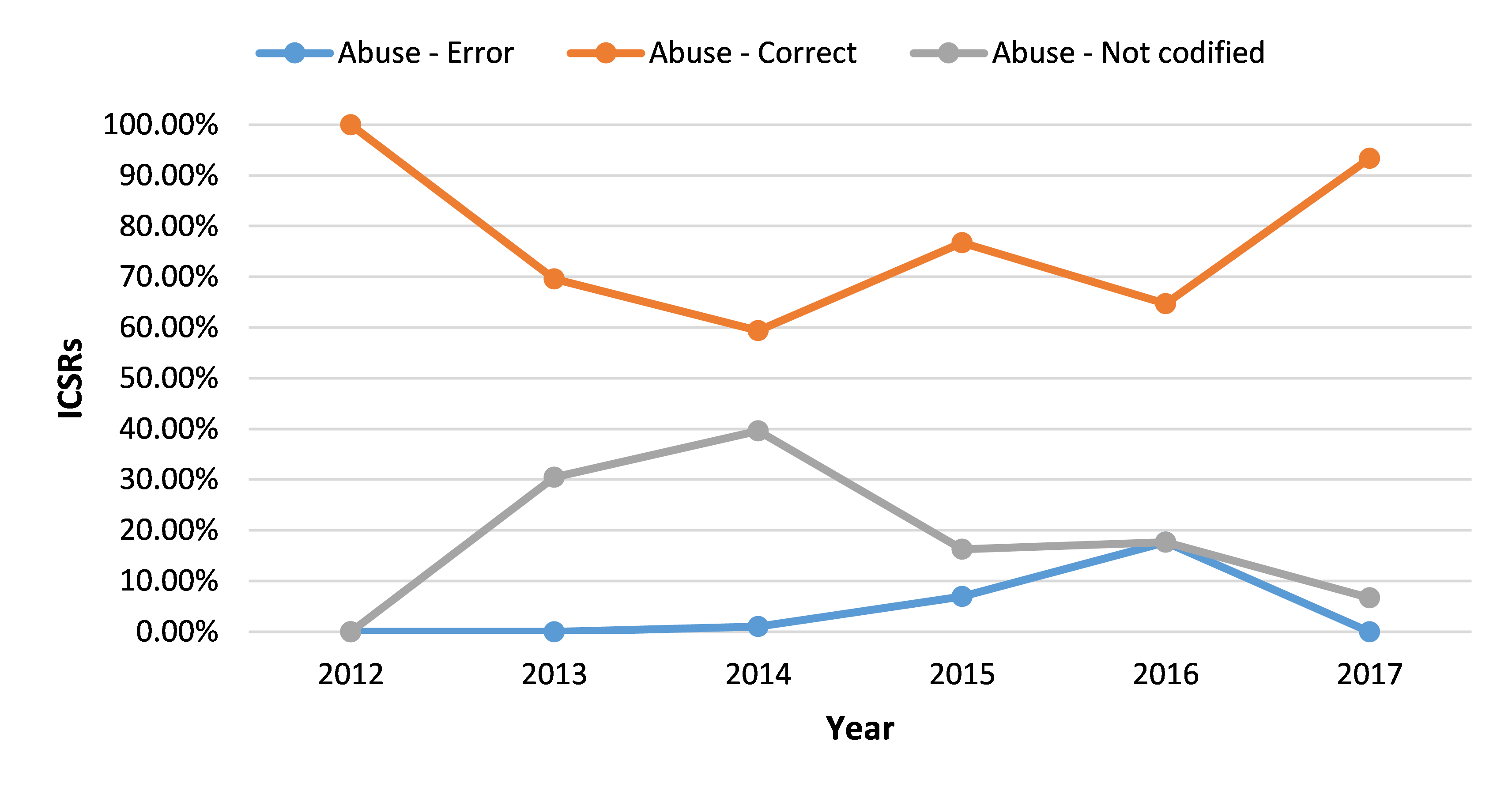

Supplement: Supplementary Figure 1 — Trend of Individual Case Safety Reports (ICSRs) deriving from abuse reported in Campania Region spontaneous reporting system from July 2nd 2012 to December 31th 2017. [file Image_1.TIF]

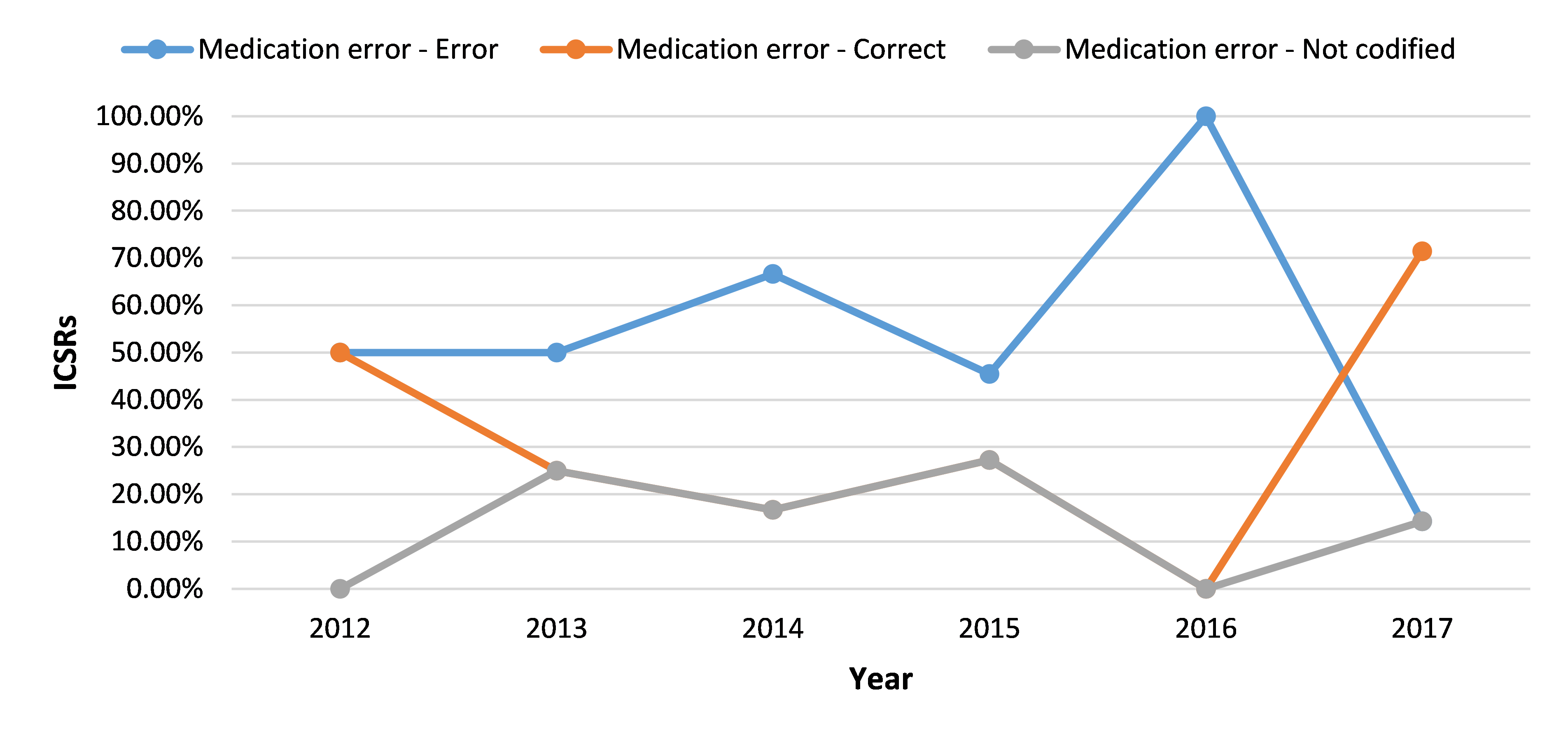

Supplement: Supplementary Figure 2 — Trend of Individual Case Safety Reports (ICSRs) deriving from medication error reported in Campania Region spontaneous reporting system from July 2nd 2012 to December 31th 2017. [file Image_2.TIF]

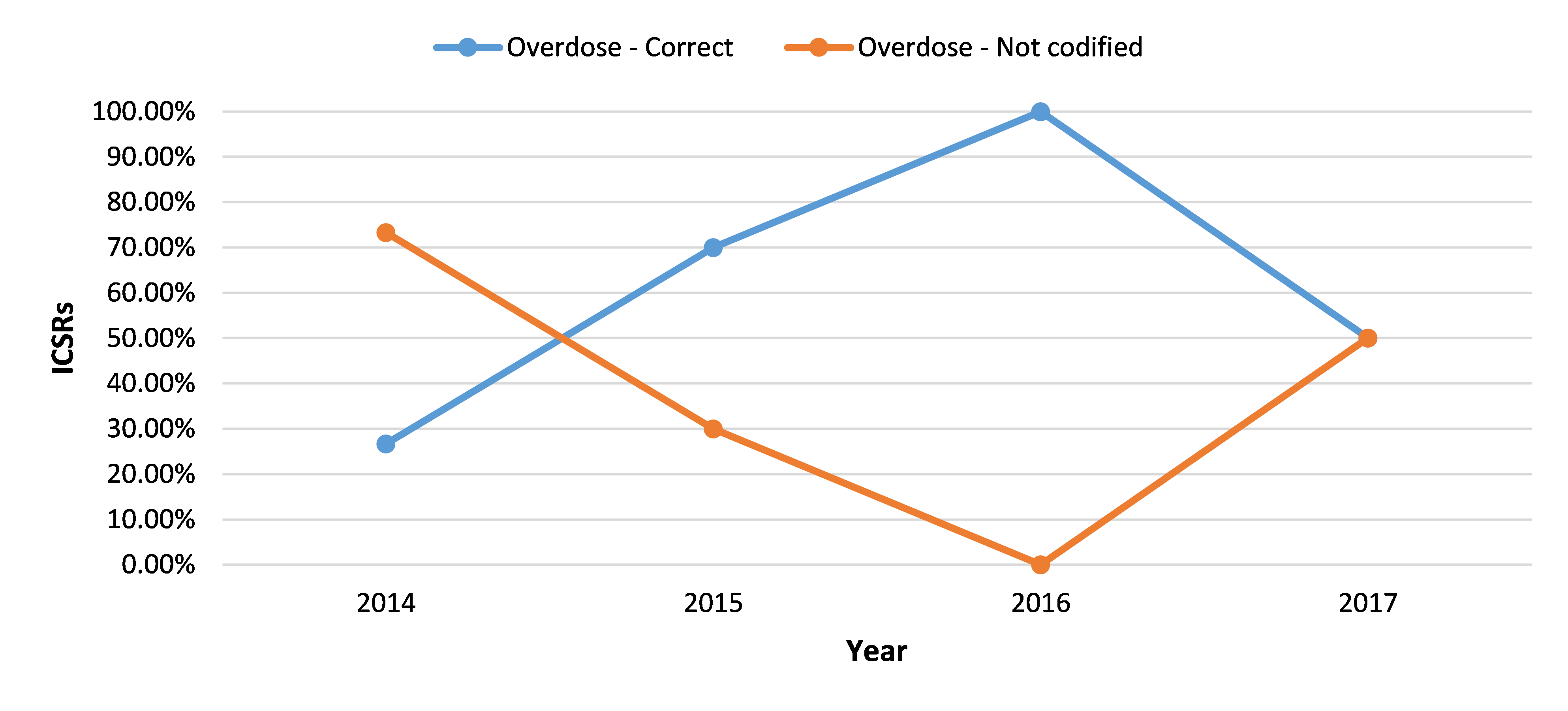

Supplement: Supplementary Figure 3 — Trend of Individual Case Safety Reports (ICSRs) deriving from overdose reported in Campania Region spontaneous reporting system from July 2nd 2012 to December 31th 2017. [file Image_3.TIF]

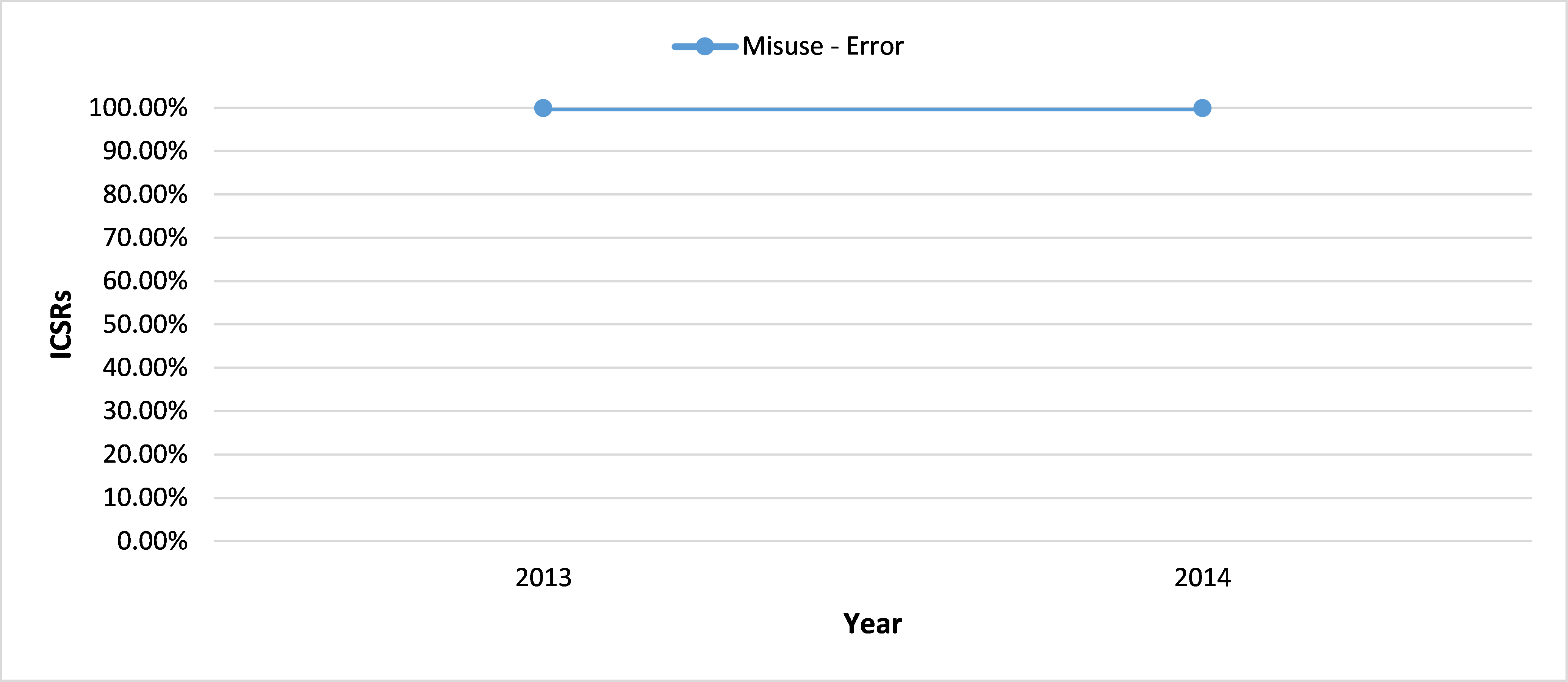

Supplement: Supplementary Figure 4 — Trend of Individual Case Safety Reports (ICSRs) deriving from misuse reported in Campania Region spontaneous reporting system from July 2nd 2012 to December 31th 2017. [file Image_4.TIF]

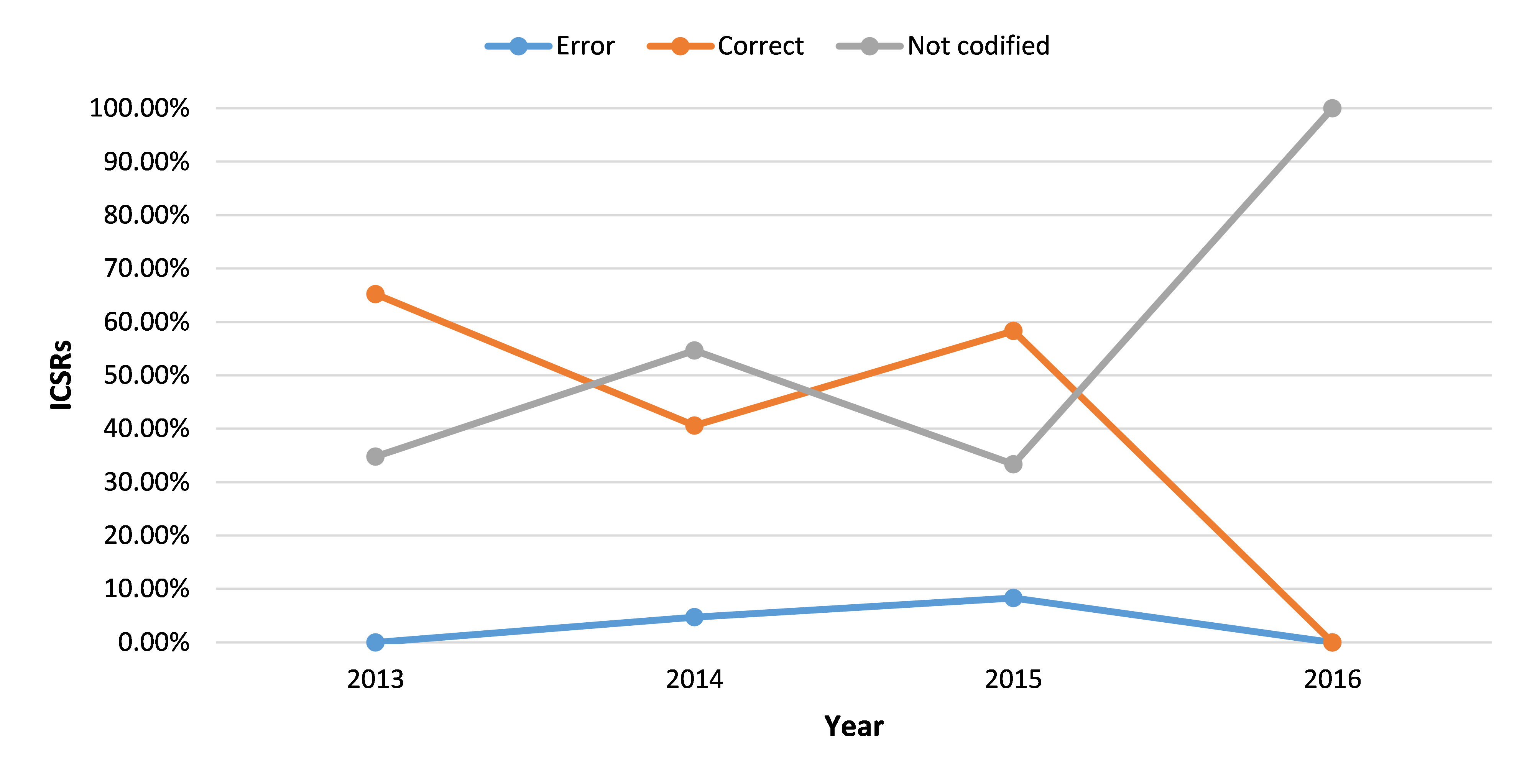

Supplement: Supplementary Figure 5 — Percentage distribution trend of un-classification or misclassification of cases reported by anti-poison center and pharmacists that were identified in Campania Region spontaneous reporting system from July 2nd 2012 to December 31th 2017. [file Image_5.TIF]
